# Pharmacological Effects of Verticine: Current Status

**DOI:** 10.1155/2019/2394605

**Published:** 2019-03-07

**Authors:** Zhenhua Yin, Juanjuan Zhang, Qingfeng Guo, Lin Chen, Wei Zhang, Wenyi Kang

**Affiliations:** ^1^Zhengzhou Key Laboratory of Medicinal Resources Research, Huanghe Science and Technology College, Zhengzhou 450063, China; ^2^Henan Joint International Research Laboratory of Drug Discovery of Small Molecules, Zhengzhou 450063, China

## Abstract

Verticine is the major bioactive constituent of* Fritillaria* as a kind of Traditional Chinese Medicine. Pharmacological researches have reported various benefits of verticine, including anticancer, anti-inflammatory, protecting against acute lung injury, tracheobronchial relaxation, antitussive, expectorant, sedative, and analgesic activities, in addition to inhibiting proliferation of cultured orbital fibroblast, angiotensin converting enzyme (ACE), and acetylcholinesterase (AChE) and inhibiting hERG potassium channels. The underlying mechanisms of verticine are still under investigation. This review will comprehensively summarize the metabolism, biological activities, and possible mechanism of verticine.

## 1. Introduction

**Figure 1:**
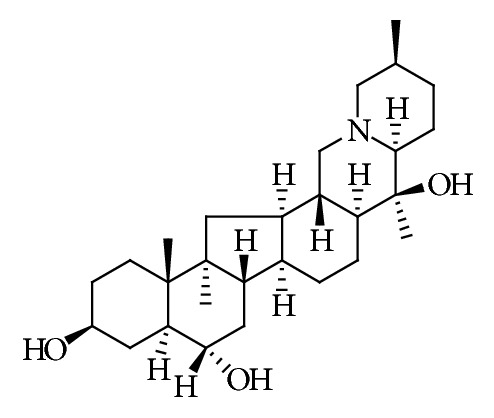
Chemical structure of verticine.

Verticine (Figure 1) belongs to a kind of isosterol alkaloid, is the major bioactive constituent of* Fritillaria* as Traditional Chinese Medicine that is widely used as an antitussive and expectorant [1]. Pharmacological researches on verticine have reported its valuable benefits in a variety of diseases, especially its anticancer effect. In this paper, the pharmacological effects, including metabolism, antitumor, anti-inflammatory, protection against acute lung injury diastolic bronchus, inhibition of angiotensin converting enzyme, and acetylcholinesterase, antitussive expectorant, sedative analgesia, were summarized, which provides theoretical references for its clinical application.

## 2. Metabolism

Pharmacokinetics of verticine is closely related to its biological activity, and the metabolism is influenced by the mode of administration, sex, and animal types. Pharmacokinetic behavior of verticine can provide the theory reference for clinical medicine.

In rabbits model, the pharmacokinetics of verticine was different between the intragastric (ig) administration and intravenous (iv) administration (Table 1). The t_1/2_ of ig administration was three times longer than that of iv administration, suggesting that there might be a reabsorption process after ig administration. However, it showed a very low bioavailability of 10.65%, which might be its low solubility in water, incomplete absorption, or metabolism of gastrointestinal enzymes and efflux pumps [2].

The pharmacokinetics of verticine was influenced significantly by sex. Verticine was eliminated slowly in the plasma of male Sprague-Dawley rat but not in female rats, and gender-related differences were also observed significantly in the pharmacokinetic parameters (Table 1). Drug concentration in blood and tissue in male rats was significantly higher except for several tissues, such as fat, muscle, and skin (data not given). Urinary cumulative excretion of verticine in female rats (0.12±0.04%) was lower than that of male rats (0.90±0.28%), and fecal cumulative excretion between female rats (0.23±0.06%) and male rats (0.27±0.06%) had no difference. Differences of sex-associated metabolism for verticine in rats are mainly due to sex-dependent expression and activity of drug metabolism enzymes and P-glycoprotein (P-gp) [3]. In addition, the main pharmacokinetic parameters of verticine were obviously different from [3] in Sprague-Dawley rats' plasma after gastric gavage extract of* Fritillaria thunbergii* Miq. The V1/F was 40.832 L/mg, indicating that verticine was mainly distributed in blood, intracellular fluid, and extracellular fluid, and it was widely distributed* in vivo* [4].

Further study showed that the intestinal absorption of verticine involved both active transport, facilitated diffusion, and resulted in the low bioavailability in male and female rats [34]. Caco-2 cell monolayer exerted an effect on the intestinal absorption of verticine. Verticine transport was of concentration-dependent type, and both *P*_app(AP-BL)_ and *P*_app(BL-AP)_ were higher at 4°C than that at 37°C. When the P-glycoprotein (P-gp) inhibitors, verapamil, and cyclosporin A were present, the *P*_app(AP-BL)_ was higher and *P*_app(BL-AP)_ was lower, and the absorption permeability was not affected by EDTA-Na_2_. The P-gp inhibitors could increase the absorption of verticine, and EDTA-Na_2_ had no discernible effect on absorption. The intestinal absorption of verticine across Caco-2 cell monolayers involved active transport rather than passive diffusion, and verticine was a substrate of P-gp [35].

## 3. Pharmacological Effects

### 3.1. Antitumor Effect

The treatment of cancer is mainly based on chemotherapy. Multiple drug resistance limited the improvement of chemotherapy efficacy and also became an important reason for the recurrence and metastasis of cancer. Verticine had the effects of anti-cell proliferation and apoptosis in many human tumor cell lines and could reverse the multidrug resistance of some drug-resistant cell lines, such as breast cancer, leukemia, lung cancer, and gastric cancer cells.

#### 3.1.1. Anti-Breast Cancer Effect

Verticine could inhibit the proliferation of breast cancer cell and induce its apoptosis and significant multidrug resistance reversal activity against breast cancer cell. Tong et al. confirmed that verticine had effects on inhibiting proliferation and reversing multidrug resistance against MCF-7/A [4]; the result was consistent with the research of Hu KW and Chen XY (1998) which confirmed that verticine showed significant multidrug resistance reversal activity against MCF-7/ADR and MCF-7 cell lines [5]. In recent years, researchers have focused on apoptosis-related protein Bcl-2. They found that Bcl-2 was involved in the tumor pathogenesis by inhibiting apoptosis. At the same time, some studies believed that the expression of Bcl-2 was one of the mechanisms of tumor resistance, and expression of Bcl-2 in tumor cells was detected during the treatment of triphenylamine (TAM) [36–38]. For breast cancer cell, Chen and Chen had proved that verticine could inhibit the proliferation of MCF -7/TAM cells (IC_50_=191.16 g/mL at 48 h and IC_50_=138.10 g/mL at 72 h) and induce its apoptosis by decreasing expression of Bcl-2 [6]. In addition, verticine showed distinct inhibiting effect on breast cancer 4T1 cells (IC_50_=14.7 *μ*mol/L at 48 h); the mechanism might be related to downregulation of TGF-*β*, VEGF, and MCP-1 secretion and decrease of TGF-*β* and VEGF mRNA expression in 4T1 cells, thereby regulating its tumor inflammatory microenvironment to show the effect of anti-cancer [7]. Further, its mechanism was investigated on 4T1 cells, and the results showed that verticine could regulate blood viscosity, improve blood flow state, reduce the expression of u-PA, VEGF, and PAI-1 protein and the secretion of IL-8, reduce the infiltration of neutrophils, improve TFPI-2 protein expression to promote tumor apoptosis, and inhibit angiogenesis and reduced cell transfer rate [8].

#### 3.1.2. Anti-Human Leukemia Effect

Verticine could inhibit the proliferation of human leukemia cell and induce apoptosis of multidrug resistant leukemia; the mechanism was likely to be related to protein expression, redox imbalance, and caspase-3. In the early studies, verticine could inhibit the proliferation of HL-60 and K562 and reverse the multidrug resistance reversal activity against HL-60/ADR and K562/A02, which might be the increase of intracellular drug concentration and inhibition of P-gp protein expression in drug-resistant cells [5, 9]. ROS was an important signal molecule in cells, involved in many events, for example, cell proliferation, apoptosis, and multidrug resistance [39, 40]. It induced ROS explosion and reduced GSH content in tumor cell to inhibit tumor cell proliferation and induce apoptosis, which had an antitumor effect [41, 42]. This finding was consistent with that of Qi et al. (2017) who proved the effect of verticine on cell viability, proliferation, and apoptosis of human leukemia and the function of reactive oxygen species and redox imbalance in this progress [10, 11]. Some alkaloids could activate caspase-3 and caspase-dependent cell apoptosis [43–45], and stimulating the ROS production of K562 cell could promote caspase-3 expression and induce apoptosis [46, 47]. Therefore, verticine might also activate caspase-3-related pathway in the process of stimulating ROS-induced apoptosis in K562/A02 cells.

#### 3.1.3. Anti-Lung Cancer Effect

Lung resistance protein (LRP) was closely related to primary resistance to cisplatin (DDP). Excision repair cross-complement 1 (ERCC1) mRNA enhanced DNA repair capacity to mediate multidrug resistance of platinum drugs [48]. Tang et al. found that verticine could inhibit A549/DDP cell proliferation in a dose-dependent manner within 48 h treatment, and the reverse index was 3.73. After 48 h, cell apoptosis rate and LRP positive cell of verticine were 38.16±2.25 and (5.8±1.3)/HP, respectively. In addition, verticine could obviously decreased the expression levels of ERCC1 mRNA and LRP. It indicated that verticine could reverse MDR of A549/DDP cell line. Its mechanism might be apoptosis induction and downregulated expression of LRP and ERCC1 mRNA [12, 13].

#### 3.1.4. Anti-Gastric Cancer Effect

Verticine could inhibit proliferation of SGC-7901 and SGC-7901/VCR, but had no obvious multiple drug resistance reversal effect [14].

### 3.2. Anti-Inflammatory Effect

Inflammation is a complex biological response mediated by activated inflammatory cells and immunocytes, involving a balance between proinflammatory and anti-inflammatory factors [15, 49]. Verticine showed anti-inflammatory effect. Zhang et al. proved that verticine could regulate inflammatory microenvironment of 4T1 breast cancer cell by controlling the release of inflammatory factors and decreasing the expression of mRNA [7]. Additionally, verticine could inhibit the gene and protein expression of MUC5AC mucin induced by EGF, PMA, or TNF-*α*, by directly acting on airway epithelial cells, and the production of MUC5AC mucin protein induced by EGF, PMA, or TNF-*α*. This finding was consistent with the traditional use of* F. thunbergii* as remedy for diverse inflammatory pulmonary diseases [16]. At the same time, Yi et al. confirmed that verticine significantly inhibited tumor necrosis factor (TNF)-*a*, interleukin (IL)-6, and IL-1*β*, increased IL-10 production in lipopolysaccharide (LPS)-stimulated RAW 264.7 macrophages, and inhibited the phosphorylation of p38, ERK and c-Jun N-terminal kinase (JNK) as well as decreased p65 and IkB, which indicated that verticine inhibited the production of inflammatory cytokines induced by LPS through blocking MAPKs and NF-*κ*B signaling pathways [50]. Verticine could inhibit the production of proinflammatory cytokines, such as IL-6, IL-8, and TNF-*α*, reducing MAPKs phosphorylation and the nuclear NF-*κ*B expression in PMACI-induced HMC-1 [17].

In addition, the activity of T cell is inhibited, which can also achieve anti-inflammatory effect; KV1.3 potassium channels play a key role in the activation of T cells. Verticine could inhibit Kv1.3 channels in a concentration-dependent manner (IC_50_=142.1 *μ*M at 150 ms) [18].

### 3.3. Protection against Acute Lung Injury

The pathogenesis of acute lung injury was complex, but its essence was the damage of lung endothelial cells and alveolar epithelial cells caused by excessive inflammation [51, 52]. LPS can activate and amplify inflammatory reactions in the body, causing the accumulation of inflammatory cells in the lungs [53]. Verticine had protective effect on LPS-induced ALI in mice; the mechanism was related to the inhibition of the inflammatory factors, the downregulation of the phosphorylation level of MAPKs in the inflammatory response signaling pathway, and the reduction of NF-*κ*B gene transcriptional intensity [19–23].

### 3.4. Tracheobronchial Relaxation and Antitussive Effects

Tracheal bronchial relaxation of verticine could be attributed to M receptor and calcium ions. Verticine showed strong inhibitory effect on the contraction of isolated tracheal strips of guinea pigs induced by carbachol; the results implied that the effect could be attributed to M receptor of the tracheal wall [24]. This result was consistent with that of Chan et al. who demonstrated the mechanisms of competitive antagonism of muscarinic pathway and also the inhibition of influx of calcium ions [25]. At the same time, verticine could also significantly elevate the concentration of cAMP in the HEK cells transfected with muscarinic M_2_ receptor plasmid [54]. However, verticine did not exhibit agonistic *β*_2_ receptor activity. It could be seen that the effect of verticine on diastolic bronchus was not produced by the agonist *β*_2_ receptor [55].

Generally, M receptor is inhibited, which could have a certain antitussive effect, and verticine is an active constituent of* Fritillaria *in relieving cough, which is related to acting on M receptor of tracheal smooth muscle to relax trachea and relieve tracheal spasm. For ammonium hydroxide induced cough in mice, mechanical stimulation of guinea pig trachea induced cough in guinea pigs, and electrical stimulation of cat superior laryngeal nerve induced cough in cats; verticine (4 mg/kg) had obvious antitussive effect. For mechanical stimulation induced cough in guinea pigs, the antitussive effect of verticine reached the peak at 30~60 min, and the antitussive effect could be sustained for about 1 h [26]. In addition, verticine could also significantly inhibit cough frequency and increase latent period of cough in mice induced by ammonia[27].

### 3.5. Expectorant and Sedative Effects

Verticine had expectorant and sedative effects. The effect was related to its ability to increase tracheobronchial mucus secretion and decrease the viscosity of mucus [27, 28]. Verticine could reduce the spontaneous activity of mice, inhibit the increasing of number of activities caused by caffeine, prolong the pentobarbital sleep time, and increase the sleep rate in mice[29].

### 3.6. Analgesic Effect

Verticine could inhibit writhing reaction induced by acetic acid in mice; at the concentration of 1 mg/kg, it had significant analgesic effect (*P*<0.05), and at the concentration of 2 mg/kg, it had very significant analgesic effect (*P*<0.01) [26]. Previous studies showed that traditional local anesthetics could play an analgesic role by nonselectively blocking the voltage-gated sodium channel subfamily, and selective Nav1.7 inhibitors were also demonstrated to be analgesic in animal models. Nav1.7 emerged as a potential target for the treatment of pain [56]. Verticine was able to block the Nav1.7 ion channel (IC_50_ = 47.2±3.3 *μ*M), which might be the analgesic mechanism of verticine [17].

### 3.7. Other Effects

Several studies showed that verticine also had other effects. Li et al. proved that verticine could inhibit the proliferation of cultured orbital fibroblast of the thyroid-associated ophthalmopathy (TAO)-patients; the inhibitory effect was obviously better than that of the normal [30]. Verticine could inhibit the activity of ACE I in a dose-dependent manner (IC_50_=312.8 *μ*M), which might be responsible, at least in part, for the antihypertensive action [31]. Unfortunately it showed no appreciable AChE inhibitory activity at a concentration of 100 *μ*g/mL, and the inhibition rate was only 25% [32]. In addition, verticine could inhibit the hERG peak tail currents with IC_50_ value of 43.7 mM, and multiple results suggested that the inhibition was related to the channel inactivation. Further investigation showed that the mechanism of the inhibition was related to the mutation of Y652 to Alanine reduced sensitivity to verticine, which suggested that Y652 was an important binding site of hERG for verticine [33]. The main concern for cardiac safety determination is the possible inhibition of hERG ion channels. So, verticine should be used with caution to avoid its toxic effect on the heart.

## 4. Conclusions

The summary of metabolism and pharmacological researches of verticine shows that the mode of administration, dose, and gender have effects on metabolism, and verticine possesses multiple pharmacological effects that are summarized in Table 2. Verticine can control the expression of related proteins, inhibit inflammatory factors, and destroy redox balance to achieve antitumor effect. The mechanism of anti-inflammatory effect was related to MAPKs and NF-*κ*B signaling pathways, and the protection against acute lung injury has close relation with anti-inflammatory effect. Acting on M receptor and inhibiting influx of calcium ions could inhibit tracheobronchial contraction, so as to play a role in relieving cough. The clinical studies of verticine remain somewhat elusive and show the risks of heart-safety.

In summary, verticine is a new potential plant-origin drug that has antitumor, anti-inflammation, protecting liver injury, and antitussive effect. The clinical effect should be focused on.

## Figures and Tables

**Table 1:** The pharmacokinetics of verticine in references.

Models	Extracts/compounds	Methods	Mode of administration	Pharmacokinetic parameters	Ref.
Rabbits	Verticine	LC-MS/MS	ig administration iv administration	A two-compartment model, C_max_= 48.31±7.40 ng/mL, AUC_0*∞*_= 270.08±80.17 ng/(mL/h), t_1/2_=6.38±3.11 h A three-compartment model, C_max_= 84.39±15.39 ng/mL, AUC_0*∞*_=50.72±14.02 ng/(mL/h), t_1/2_= 2.19±1.07 h	[2]

Sprague-Dawley female rats Sprague-Dawley male rats	4.25 g/kg Fritillaria thunbergii Miq. Extract	LC-MS/MS	a single oral administration	C_max_= 43.7±22.7 ng/mL, T_max_=1.5±0.7h, t_1/2_=4.2±2.0 h, AUC_0t_= 214.2±84.6 ng/(mL/h), AUC_0*∞*_= 214.3±84.5 ng/(mL/h), CL/F= 128.9±32.6 L/kg/h, V/F= 781.3±305.6 L/kg C_max_= 57.6±21.6*∗* ng/mL, T_max_=2.9±1.7*∗* h, t_1/2_=6.2±1.9 h, AUC_0t_= 662.4±277.9*∗* ng/(mL/h), AU_C0*∞*_= 665.3±213.3*∗* ng/(mL/h), CL/F= 41.5±20.1 L/h/kg, V/F= 374.1±186.2 L/kg	[3]

Sprague-Dawley Rats	Fritillaria thunbergii Miq. Extract (0.45 g/kg body weight)	RRLC-MS/MS	gastric gavage	A two-compartment model, C_max_= 3.671±0.876 *μ*g/L, T_max_=32.5±11.292 min, t_1/2*α*_= 29.269±24.156 min, t_1/2*β*_=162.897±30.669 min, AUC_0t_= 628.568±100.99 *μ*g/(L*∗* min), AUC_0*∞*_= 630.875±102.136 *μ*g/(L*∗* min), CL/F= 0.423±0.075 L/min/kg, V1/F= 40.832±17.616 L/kg	[4]

^*∗*^ *p*<0.05, significantly different from female rats.

**Table 2:** The biological activities of verticine in references.

Activities	Models	Biological activities	Action mechanism	Ref.
Anti-breast cancer	MCF-7	Inhibit proliferation, reversing multidrug resistance	-	[4, 5]
	MCF -7/TAM	Inhibit proliferation (at 48 h IC_50_=191.16 g/mL; at 72 h, IC_50_=138.10 g/mL), induce apoptosis	Decrease expression of Bcl-2	[6]
	4T1	Inhibit proliferation (at 48h, IC50=14.7 *μ*mol/L)	(1) Down-regulate TGF-*β*, VEGF and MCP-1 secretion, decrease TGF-*β* and VEGF mRNA expression, regulating its tumor inflammatory microenvironment. (2) Regulate blood viscosity, improve blood flow state, reduce the expression of u-PA, VEGF, PAI-1 protein and the secretion of IL-8, reduce the infiltration of neutrophils, improve TFPI-2 protein expression	[7, 8]

Anti-human leukemia	HL60, HL-60/ADR, K562 K562/A02	Inhibit proliferation (IC_50_=288.27±34.23, 256.52±26.15, 320.80±36.52, 300.06±33.18, *μ*g/mL), reverse multidrug resistance	Increase intracellular drug concentration and inhibit P-gp protein expression	[5, 9]
	K562 /A02	Inhibit the cell viability and induce apoptosis, different concentrations of verticine (100, 200, 400 mol/L); the cell viabilities were 0.392±0.040, 0.314±0.022, 0.161±0.033	Induce the ROS outbreak and increase the GSH content, redox imbalance	[10, 11]
	K562	Inhibit proliferation (IC_50_=238 mol/L)		

Anti-lung cancer	A549/DDP	Inhibit proliferation, induce apoptosis, reversing multidrug resistance	Down-regulate expression of LRP and ERCC1 mRNA	[12, 13]

Anti-gastric cancer	SGC-7901 and SGC-7901/VCR	Inhibit proliferation	-	[14]

Anti-inflammatory effect	4T1	Regulate inflammatory microenvironment	Control release of inflammatory factors, such as TGF-*β*, VEGF, MMP-9, and MCP-1, decreasing the expression of TGF-*β* and VEGF mRNA	[6]
	Confluent NCI-H292 cells	Remedy for inflammatory pulmonary diseases	Inhibit gene and protein expression of MUC5AC mucin induced by EGF, PMA or TNF-*α* by directly acting on airway epithelial cells	[15]
	LPS-induced RAW264.7 macrophages	Inhibit production of inflammatory cytokines induced by LPS	Block MAPKs and NF-kB signaling pathways	[16]
	HMC-1 Cells	Inhibit production of inflammatory cytokines	Regulate the Phosphorylation of NF-*κ*B and MAPKs	[17]
	HEK 293	anti-inflammatory	Inhibit Kv1.3 channels	[18]
Protection against acute lung injury	Mice	Protective effect on acute lung injury	Inhibit expression of TNF-*α*, IL-2, IL-6 and IL-8 and COX-2, promote the synthesis and release of SP-A, decrease the levels of PGE2 and NO. And, in addition, down-regulate phosphorylation level of MAPKs in the inflammatory response signaling pathway, and reduce NF-*κ*B gene transcriptional intensity	[19–23]

Tracheobronchial Relaxation	Isolated tracheal strips of guinea pigs	Inhibit contraction	M receptor	[24]
	rat isolated tracheal and bronchial	Inhibit carbachol-induced contraction	Inhibit influx of calcium ions	[25]

Antitussive effect	Mice	Antitussive effect for ammonium hydroxide induced cough (4 mg/kg)	-	[26]
	Guinea pig trachea	Antitussive effect for mechanical stimulation induced cough(4 mg/kg)	-	[26]
	Cat superior laryngeal nerve	Antitussive effect electrical stimulation induced cough (4 mg/kg)	-	[26]
	Mice	Inhibit cough frequency and increase latent period of cough induced by ammonia	-	[27]

Expectorant effect	Mice's tracheal	Enhance mice's tracheal phenol red output in expectorant evaluation	Increase tracheobronchial mucus secretion and decrease the viscosity of mucus	[27, 28]

Sedative effect	Mice	Reduce spontaneous activity, prolong pentobarbital sleep time and increase sleep rate	-	[29]

Analgesic effect	Mice	Inhibit writhing reaction induced by acetic acid	Block Nav1.7 ion channel (IC_50_=47.2±3.3 *μ*M)	[17, 26]

Inhibit proliferation of cultured Orbital fibroblast	Thyroid-associated ophthalmopathy (TAO)-patients	Different concentrations (5, 25,50, 75, 100 mg/L) inhibit the proliferation of orbital fibroblasts in vitro in a dose-dependent manner.	-	[30]

Inhibit angiotensin converting enzyme (ACE) activity	Rat plasma	Inhibit ACE I activity in a dose-dependent manner (IC_50_=312.8 *μ*M)	-	[31]

Inhibit acetylcholine (AChE) inhibitory activity		At 100 *μ*g/mL, inhibition rate was only 25%	-	[32]

Inhibit hERG potassium channels	HEK293 cell line	Inhibit hERG peak tail currents with IC_50_ value of 43.7 mM	channel inactivation, Mutation of Y652 to Alanine reduced sensitivity	[33]
